# Outcome Improvement in RPA I or II Patients With 1 or 2 Brain Metastases by Combined Surgery and Radiotherapy

**DOI:** 10.4021/wjon626w

**Published:** 2013-03-06

**Authors:** Delphine Antoni, Pierre Kehrli, Jean-Baptiste Clavier, Mohamed Amine Lahlou, Francois Lefebvre, Georges Noel

**Affiliations:** a Radiotherapy Department, Centre de Lutte Contre le Cancer Paul Strauss, 3, rue de la Porte de l’hopital, 67065 Strasbourg cedex, France; b Neurosurgical Department, Hopital de Hautepierre, 1, Avenue Moliere, 67098 Strasbourg cedex, France; c Laboratory of Biostatistics, Faculte de Medecine, 4, rue Kirschleger, 67085 Strasbourg cedex, France

**Keywords:** Brain metastases, Whole brain radiotherapy, Surgery

## Abstract

**Background:**

To evaluate the role of surgery and postoperative radiotherapy in the management of brain metastases (BM): a retrospective analysis for overall survival (OS), local and brain control (LC and BC) of a series of 329 patients with recursive partitioning analysis (RPA) I or II with 1 or 2 BM in a single institution.

**Methods:**

Patients were treated either with combined surgical resection and whole brain radiation therapy (WBRT) in 104 cases (31.6%) or with WBRT alone in 225 cases (68.4%). Ninety-five patients (91.4%) who underwent surgery and WBRT and 147 (65.3%) who underwent WBRT alone benefited from a radiation boost to the metastatic site.

**Results:**

The median OS was higher for patients RPA I compared to RPA II: 21.3 and 5.9 months (P < 0.0001), as well as for the surgical group compared to the radiation group: 20.2 vs 5.3 months (P < 0.0001), respectively. After the multivariate analysis, the improved OS was significantly associated with control of primary tumor (P = 0.0002) after surgical resection and with type of primary tumor (P = 0.002), absence of extracranial metastases (ECM) (P = 0.006), and high Karnofsky performance status (90 - 100 vs 70 - 80) (P = 0.003) after radiotherapy alone. The 12-, 24- and 36-months LC rates were 91.1%, 91.1% and 83.9%, respectively, after surgical resection and 81.2%, 63.1% and 57.3%, respectively, after radiotherapy alone (P = 0.005). In a univariate analysis, improved LC for the surgical group was also associated with the absence of ECM (P = 0.01) and for the radiation group, with a radiation boost (P = 0.01). The BC rates at 12, 24 and 36 months were 73.2%, 66.9% and 56%, respectively, in the surgical group and 75.7%, 49.6% and 42.4%, respectively, in the radiation group (P = 0.2). In our univariate analysis, improved BC after surgical resection was associated with control of primary tumor (P = 0.02). For patients in the radiation group, gender (P = 0.03) and a radiation boost (P = 0.0003) were significant prognostic factors in a univariate analysis. In our multivariate analysis, only the radiation boost was significant (P = 0.001).

**Conclusions:**

Surgical resection followed by WBRT leads to a better outcome compared to WBRT alone for RPA I or II patients with 1 or 2 BM.

## Introduction

Despite improved treatment of systemic diseases and efficient methods of imaging, brain metastases (BM) remain a clinical and therapeutic challenge. Surgery remains essential to obtain the histology of the primary tumor. For metastases up to 3 centimeter in size, surgery is also essential for a better prognosis and increased survival. Surgery seems to improve the outcome of patients, but still remains controversial [1-5]. Even if the resection is complete, rates of locoregional relapse after surgery alone can reach 85% [6]. Whole brain radiation therapy (WBRT) after surgical resection results in improved survival compared to surgery or WBRT alone. Patchell et al randomized 48 patients with a single BM into groups of either surgical resection followed by WBRT (n = 25) and WBRT alone (36 Gy in 12 fractions, 3 Gy per fraction) (n = 23) [1]. The overall length of survival was significantly longer in the surgical group (median, 40 weeks vs. 15 weeks in the radiation group; P < 0.01). The patients treated with surgery remained functionally independent for longer (median, 38 weeks vs 8 weeks in the radiation group; P < 0.005). Recurrence at the site of the original metastasis was also less frequent in the surgical group than in the radiation group (20% vs. 52%; P < 0.02). The purpose of this study was to evaluate the role of surgery and postoperative radiotherapy in the management of 1 or 2 BM for RPA (recursive partitioning analysis) I or II patients in a large series.

## Methods and Materials

### Study design and patient population

The present study was a single institution retrospective analysis of a database of 329 patients treated for BM between September 2005 and December 2010 at the Center Paul Strauss. The data from patients with RPA I or II with 1 to 2 resectable BM treated with different schedules was analyzed. For patients who underwent surgery, the median interval between BM diagnosis and surgical resection was 12.5 days (0 - 909 days), and the median interval between surgical resection and radiotherapy was 41 days (9 - 497 days). In total, the median interval between BM diagnosis and radiotherapy was 62.5 days (8 - 941 days). For patients who were treated with radiotherapy alone, the median interval between BM diagnosis and WBRT was 39 days (1 - 1,005 days) (P < 0.0001).

All of the patients who underwent surgery did not receive a previous biopsy. Surgery was considered complete by the surgeons according to their assessment. None of the patients were re-operated on due to side effects. For the irradiation patients, a CT scan was performed for dosimetry. A custom plastic mask was used for treatment. WBRT was performed in all of the patients with 6 MV photons of a linear accelerator, using bilateral fields. The radiation boost on the operative site was delivered with 6 or 25 MV, using 2 or three fields, after 3-dimensional treatment planning. For RPA I patients, a dose of 40 Gy in 20 fractions of 2 Gy (5 fractions per week) was delivered in the whole brain. A radiation boost on the operative site was delivered in a dose of 16 Gy in 8 fractions of 2 Gy (5 fractions per week) after surgical resection. For RPA class II patients, a dose of 30 Gy in 10 fractions of 3 Gy (5 fractions per week) was delivered in the whole brain. A radiation boost at the operative site was delivered at a dose of 9 Gy in 3 fractions of 3 Gy (3 fractions per week) after surgical treatment. For RPA I patients who benefited only from WBRT, a dose of 37.5 Gy in 15 fractions of 2.5 Gy (5 fractions per week) was delivered followed by a boost at the metastatic site (10 Gy in 4 fractions of 2.5 Gy, 4 fractions per week). For RPA II patients, WBRT was delivered at 30 Gy in 10 fractions of 3 Gy (5 fractions a week) followed by a boost of 9 Gy (in 3 fractions of 3 Gy, 3 fractions a week).

### Statistical analysis

The primary endpoint for these analyses was overall survival (OS) from the last day of radiotherapy. The secondary endpoints were local control (LC) and brain relapse-free survival (BFS) (the absence of local and regional failure or relapse). The time to any endpoint was measured from the date of radiotherapy completion. All of the patients alive at the time of analysis were censored according to the date of their last follow-up. The survival probabilities were calculated using the Kaplan-Meier method for each of the subgroups. The subgroups were defined by the BM treatment type and were divided into the surgical group (surgery followed by WBRT) and the radiation group (WBRT alone). Nine potential prognostic factors were evaluated with respect to the overall survival, local control and brain control and the type of treatment. These factors were gender, the Karnofsky performance status (KPS), the primary tumor type, the presence of ECM, the number of ECM, control of the primary tumor, a radiation boost at the metastatic site, the interval between BM diagnosis, the treatment and the interval between BM diagnosis and surgical resection. All P values < 0.05 were considered statistically significant.

## Results

There were 223 men (67.8%) and 106 women (32.2%) in our study. The patients (with a median age 61.4 at diagnosis) were treated either with surgical resection followed by brain radiotherapy or with brain radiotherapy alone in 104 (31.6%) and 225 cases (68.4%), respectively. The patient characteristics are summarized in Table 1. The two groups were comparable except for age (the patients were older in the radiation group, P = 0.02) and distribution of the primary tumor (P = 0.002). Ninety-five patients (91.4%) who underwent surgery and 147 (65.3%) who benefited from WBRT alone received a radiation boost at the metastatic site. The diagnosis was established by a contrast CT scan (45.9%), MRI (17.0%) or both (36.8%) (for one patient we did not have the information). The BM was located in the cerebral hemispheres (76.6%), in the cerebellum (16.1%) or in both sites (7.3%). The primary tumors were lung (n = 205, 62.3%), breast (n = 33, 10%), gastrointestinal (n = 27, 8.2%), melanoma (n = 20, 6.1%), kidney (n = 16, 4.9%) and other sites (n = 28, 8.5%). In the surgical group, the patients were RPA I and II in 46 and 58 cases (44.2% and 55.8%), respectively, and had 1 or 2 BM in 94 and 10 cases (90.4% and 9.6%), respectively. In the radiation group, the patients were RPA I and II in 17 and 208 cases (7.6% and 92.4%), respectively, and had 1 or 2 BM in 133 and 92 cases (59.1% and 40.9%), respectively (the differences were not significant). In 84 cases, the patients had a complete metastatic resection (80.8%). Five patients had a partial resection (4.8%), and there was no information available for the remaining 15 patients (14.4%).

**Table 1:** Patient Characteristics

Characteristic	Value			P
	Entire series (n = 329) No (%)	Surgical group (n = 104) No (%)	Radiation group (n = 225) No (%)	
Age (y)				0.02
Median	61.4 (20.9 - 86.2)	58.5 (38.6 - 78.3)	63.8 (20.9 - 86.2)	
Gender (n)				0.8
Male	223 (67.8)	59 (56.7)	164 (72.9)	
Female	106 (32.2)	45 (43.3)	61 (27.1)	
KPS (n)				0.61
70 - 80	203 (61.7)	50 (48.1)	153 (68.0)	
90 - 100	126 (38.3)	54 (51.9)	72 (32.0)	
BM (n)				0.9
1	227 (69.0)	94 (90.4)	133 (59.1)	
2	102 (31.0)	10 (9.6)	92 (40.9)	
ECM (n)				0.9
Yes	217 (66.0)	47 (45.2)	170 (75.6)	
No	112 (34.0)	57 (54.8)	55 (24.4)	
Number of ECM (n)				0.33
1	101 (46.5)	33 (70.2)	68 (40.0)	
≥ 2	116 (53.5)	14 (29.8)	102 (60.0)	
Control of primary tumor (n)				0.9
Yes	141 (42.9)	54 (51.9)	87 (38.7)	
No	188 (57.1)	50 (48.1)	138 (61.3)	
Neurological symptoms (n)				0.86
Yes	204 (62.0)	82 (78.8)	122 (54.2)	
No	125 (38.0)	22 (21.2)	103 (45.8)	
Boost (n)				0.28
Yes	242 (73.6)	95 (91.4)	147 (65.3)	
No	87 (26.4)	9 (8.6)	78 (34.7)	
RPA (n)				0.75
Class I	63 (19.1)	46 (44.2)	17 (7.6)	
Class II	266 (80.9)	58 (55.8)	208 (92.4)	
Site of primary tumor (n)				0.002
Lung	205 (62.3)	56 (53.8)	149 (66.2)	
Breast	33 (10.0)	13 (12.5)	20 (8.9)	
Melanoma	20 (6.1)	9 (8.7)	11 (4.9)	
GI	27 (8.2)	10 (9.6)	17 (7.6)	
RCC	16 (4.9)	9 (8.7)	7 (3.1)	
Other	28 (8.5)	7 (6.7)	21 (9.3)	

KPS: karnofsky performance status; BM: brain metastases; ECM: extracranial metastases; RPA: recursive partitioning analysis; GI: gastrointestinal; RCC: renal cell carcinoma.

### Overall survival

In the two groups, 49 patients (14.9%) were still alive at the time of analysis and 280 (85.1%) were dead. Neurological death occurred in 40 patients (14.3%); in 140 patients (50%), death was attributed to another cause; and in 100 patients (35.7%), the cause was unknown or the information not available. In the surgical group, 37 patients (35.6%) were still alive at the time of analysis and 67 patients (64.4%) were dead. Neurological death occurred in 14 patients (20.9%); in 31 patients (46.3%), death was attributed to another cause; and in 22 patients (32.8%), the cause was unknown. In the radiation group, only 12 patients (5.3%) were alive at the time of analysis and 213 were dead (94.7%). Twenty-six deaths (12.2%) were attributed to a neurological cause, 109 (51.2%) were not, and 78 patients (36.6%) died of an unknown cause. The difference in terms of neurological death between the two groups was not significant (P = 0.08). The median survival rate in the surgical and radiation alone groups was 20.2 and 5.3 months, respectively. In the surgery group, the 12-, 24- and 36-month OS rates were 62.1%, 46.5% and 32%, respectively. In the radiation alone group, the 12-, 24- and 36-month OS rates were 27.1%, 10.5% and 6.1%, respectively (P < 0.0001) (Table 2).

**Table 2:** Results of Local Control, Brain Control and Overall Survival of the 2 Treatment Groups

	At 6 months (%)	At 12 months (%)	At 18 months (%)	At 24 months (%)	At 36 months (%)	P
Local control						
Surgical group	92.7	91.1	91.1	91.1	83.9	0.005
Radiation group	88.9	81.2	72.6	63.1	57.3	
Brain control						
Surgical group	87	73.2	68.9	66.9	56	0.2
Radiation group	86.2	75.7	63	49.6	42.4	
Overall survival						
Surgical group	75.7	62.1	52.5	46.5	32	< 0.0001
Radiation group	45.6	27.1	17.8	10.5	6.1	

In the surgical group, after univariate analysis, improved OS was significantly associated with the absence of ECM (P = 0.03) and control of primary tumor (P < 0.0001). In multivariate analysis, OS was significantly associated with control of the primary tumor (HR 0.3, 95% CI 0.2 - 0.6, P = 0.0002).

After a univariate analysis, the radiation alone group showed an improved OS, which was associated with gender (P = 0.0006), the type of primary tumor (P = 0.0001), the absence of ECM (P = 0.01), control of the primary tumor (P = 0.04), a high KPS (P = 0.001) and a radiation boost at the metastatic site (P = 0.01). After a multivariate analysis, the favorable significant prognostic factors were the absence of ECM (HR 1.6, 95% CI 1.14 - 2.29, P = 0.006) and a high KPS (KPS 90 - 100 vs 70 - 80) (HR 0.63, 95% CI 0.46 - 0.85, P = 0.003). In this analysis, the type of primary tumor (P = 0.002) was also significant.

### Median survival by RPA class and treatment

**Figure 1:**
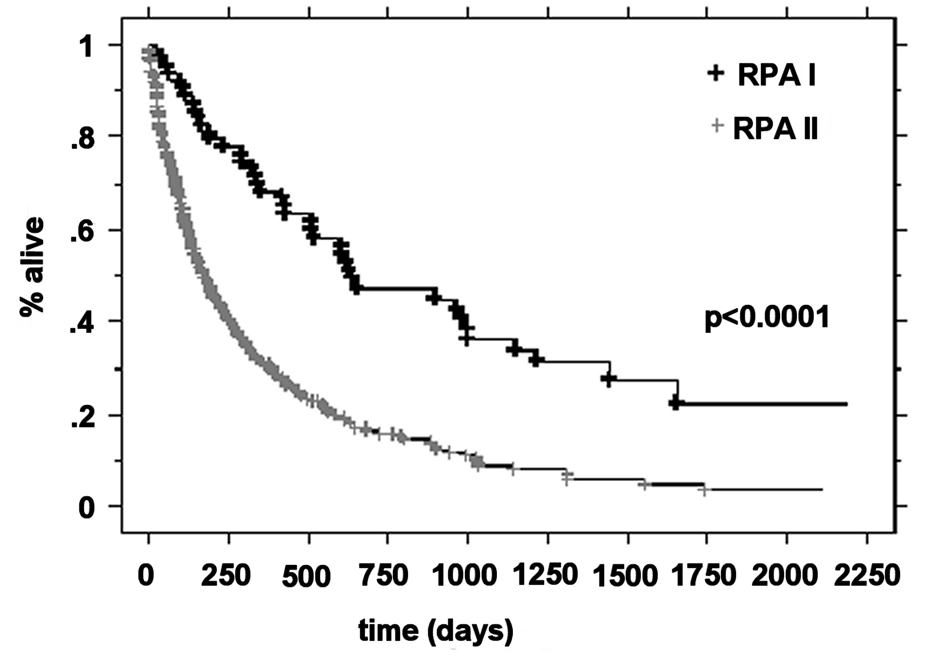
Kaplan-Meier curves for the overall survival time for RPA class.

**Figure 2:**
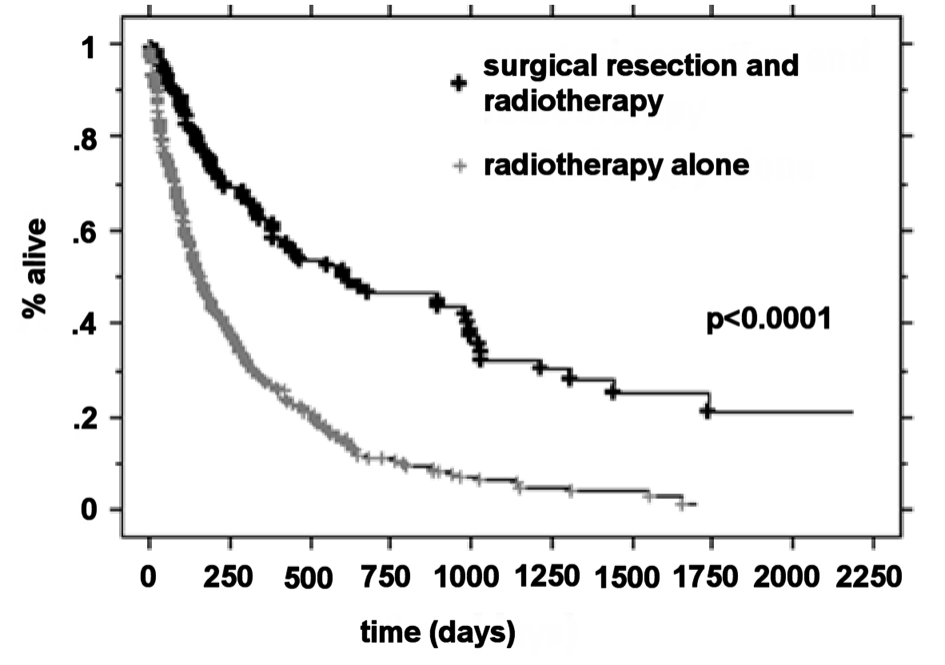
Kaplan-Meier curves for the overall survival time for treatment.

**Figure 3:**
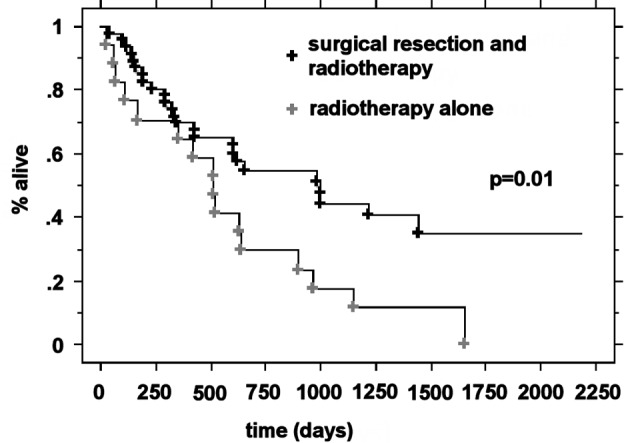
Kaplan-Meier curves for the overall survival time for RPA class I patients and treatment.

**Figure 4:**
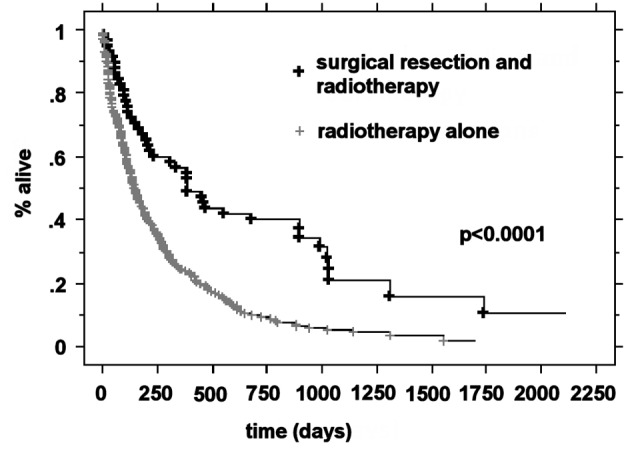
Kaplan-Meier curves for the overall survival time for RPA class II patients and treatment.

The median OS was higher in the RPA I group compared to the RPA II group at 21.3 months (n = 63) and 5.9 months (n = 266), respectively (P < 0.0001). Patients with RPA I or II who underwent surgical resection had improved outcomes compared to those who were treated with radiotherapy alone. The median survival for these two groups was 20.2 months and 5.3 months, respectively (P < 0.0001). For RPA I patients, there was a significant improvement in the overall survival in the surgical group compared to the definitive WBRT group (33.2 months vs. 17.2 months, P = 0.01). RPA II patients who underwent surgery presented with significantly improved outcomes compared to the RPA II patients treated with radiotherapy alone (13 months vs. 4.8 months, respectively P < 0.0001) (Fig. 1-4).

### Local and brain relapse-free survival

In the surgical group, a recurrence anywhere in the brain occurred in 35 patients (33.6%), with a median appearance time of 10.1 months (range 1 - 46 months). Local recurrence, regional recurrence or both were observed for 7 patients (20%), 25 patients (71.4%) and 3 patients (8.6%), respectively. In the radiation alone group, a recurrence anywhere in the brain (local or distant intracerebral failure) during the follow-up period occurred in 47 patients (20.9%), with a median appearance time of 7.6 months (range 1 - 54 months). Local recurrence, regional recurrence or both occurred for 22 patients (46.8%), 15 patients (31.9%) and 9 patients (19.2%), respectively (Table 3).

**Table 3:** Brain, Local and Regional Recurrences According to Treatment

	Brain recurrence n	Local recurrence n (%)	Regional recurrence n (%)	Both n (%)	Unknown n (%)
Surgical group (n = 104)	35	7 (20)	25 (71.4)	3 (8.6)	
Radiation group (n = 225)	47	22 (46.8)	15 (31.9)	9 (19.2)	1 (2.1)
Total (n = 329)	82	29 (35.4)	40 (48.8)	12 (14.6)	1 (1.2)

### Local control

In the surgical group, the 12-, 24- and 36-month LC rates were 91.1%, 91.1% and 83.9%, respectively. In the radiation alone group the 12-, 24- and 36-month LC rates were 81.2%, 63.1% and 57.3%, respectively (P = 0.005).

In the surgical group, using univariate analysis, improved LC was significantly associated with the absence of extracranial metastases (P = 0.01).

In the radiation alone group, using univariate analysis, only the radiation boost at the metastatic site was a significant prognostic factor of LC (P = 0.01).

### Brain control

In the surgical group, the 12-, 24- and 36-month BFS rates were, 73.2%, 66.9% and 56%, respectively. In the radiation alone group, the 12-, 24- and 36-month BFS rates were 75.7%, 49.6% and 42.4%, respectively (P = 0.2).

In the surgery group, using univariate analysis, we found that improved BFS was also significantly associated with control of the primary tumor (P = 0.02).

In the radiation alone group, using univariate analysis, gender (P = 0.03) and a radiation boost at the metastatic site (P = 0.0003) were significant prognostic factors of BFS. Using multivariate analysis, only the radiation boost was a favorable significant prognostic factor for BFS (HR 2.63, 95% CI 1.45 - 4.77, P = 0.001).

## Discussion

Even though our study is retrospective, it possesses several strengths. Our study utilized a large series of RPA I or II patients with 1 or 2 BM treated homogeneously in a single institution, according a guideline several region in France. In our series, combined therapy significantly improved the overall survival and the local control rates compared to radiotherapy alone. The brain control rate was also improved however; the difference was not statistically significant. Our results can be favorably compared with the results of other randomized trials. The addition of WBRT to local treatment in oligometastatic brain disease leads to a better outcome compared to WBRT or surgery alone. Only one randomized trial [4] has been performed where the resection and the WBRT group demonstrated a significant decrease in tumor recurrence locally (10% vs 46%) and elsewhere in the brain (18% versus 70%) compared to the group treated with surgical resection alone. The EORTC 22952-26001 study showed that after surgery to remove a limited number of BM, adjuvant WBRT reduced the 2-year relapse rate both at the initial sites (59% to 27%, P < 0.001) and at new sites (42% to 23%, P = 0.008) and also reduced neurologic deaths [5]. Additionally, the addition of local treatment to WBRT leads to a better outcome compared to WBRT alone. In the surgery group in our study, the 2-year local and brain relapses rates were 8.9% and 33.1%, respectively. Moreover, our study shows an improved outcome in terms of overall survival for patients treated with combined therapy compared to radiotherapy alone. Patchell et al [1] performed a randomized clinical trial, which compared surgical resection plus WBRT (n = 25) to WBRT alone (n = 23) for single brain metastases. This study demonstrated a significant overall survival advantage in the surgery plus WBRT group (40 weeks) compared to the WBRT alone group (15 weeks). Vecht et al [3] randomized 63 patients with single BM into two groups (surgery plus WBRT vs WBRT alone). The overall survival was 10 months in those treated with surgery plus WBRT compared to 6 months for WBRT alone. Our median survival rate was comparable to these previously published results. However, other trials failed to demonstrate a significant difference in OS [7, 8]. The difference of RPA distribution between the two groups in our series can explain the OS improvement in the surgery group. This explanation remains partial because the differences in the cause of death were not significantly different between the groups. In a retrospective study, Rades et al compared 99 RPA I or II patients treated for 1 or 2 BM. In this study, some patients received surgical resection and WBRT and 102 patients received surgical resection followed by WBRT and a radiation boost [9]. Based on this retrospective series, our study is also comparable in terms of patient distribution. Our results are in accordance with the rates of overall survival, local and brain control for patients treated by surgical resection, WBRT and radiation boost. The 12-, 18- and 24-month OS rates were 66%, 51% and 48%, respectively, for Rades et al and 62.1%, 52.5% and 46.5%, respectively, in our study. The 12-, 18- and 24-month BFS rates were 71%, 60% and 50%, respectively, for Rades et al and 73.2%, 68.9% and 66.9%, respectively, in our study. The LC rates were improved in our study compared to the study by Rades et al. The fact that metastatic resection was complete in 80.8% of cases in our series, more than in the series of Rades et al (64%), could explain this difference. Moreover, the extent of surgical resection was a significant prognostic factor in the univariate and multivariate analysis for Rades et al. In our series, RPA I patients had an improved outcome compared to RPA II patients. RPA I patients had a better median overall survival than the 1200 patients from three RTOG trials conducted between 1979 and 1993 [10] (21.3 months vs. 7.7 months). Also, RPA II patients in our series had an improved outcome compared to those in the RTOG trials (5.9 months vs. 4.7 months, respectively). However, in the three RTOG trials patients were treated with radiation therapy alone. Furthermore, RPA I patients treated with radiation therapy alone in our series had an improved outcome compared to those patients in the RTOG trials (17.2 months vs. 7.7 months, respectively). However, in our series RPA patients I had only 1 or 2 BM. Therefore, the RTOG 9508 demonstrated a better outcome for patients with one site of metastasis, compared to those with two or three BM [11]. For RPA II patients, the results were similar (4.8 months vs. 4.7 months, respectively).

The results of radiosurgery are always compared to surgery. In the EORTC trial 22952-26001, the results of surgery alone, radiosurgery alone, surgery plus WBRT and radiosurgery plus WBRT were not different [5]. In retrospective studies the results are debatable [12, 13]. According to the results of our studies, surgery plus WBRT is more useful in terms of outcome improvement compared to WBRT alone, mainly when radiosurgery is not available. Another interesting development in radiosurgery is the use of radiation in the surgery bed alone to reduce the risk of cognitive impairment arising after WBRT. Some studies positively argued for this treatment option [14]. In our institution, the radiosurgery is available since 2010, and this therapeutic alternative is used for patients with one to three BM less than 3 centimeters and for radiation in the surgery bed. These patients are regularly followed (clinical examination and Magnetic Resonance Imaging): at one month after completion of radiotherapy, every 4 months the first year, every six months the second year and once a year from the third year. In case of regional relapse, another radiosurgery or WBRT would be discussed. However, even if this treatment can prevent local relapse, the risk of brain new metastases remains challenging. Without WBRT, the risk of brain relapse is approximately 50%. With WBRT, the risk relief is approximately 25%. In total, WBRT seems to benefit only 25% of the patients. Among these patients, some can be treated by surgery or radiosurgery again, postponing the use of WBRT. Randomized trials are required to reach further definitive conclusions.

### Conclusion

Our study underlines the role of surgery, which remains a cornerstone in the treatment of brain metastases. Surgical resection followed by radiation treatment leads to a better outcome compared to radiotherapy alone for patients with 1 or 2 BM, and for RPA I or II, in terms of overall survival, brain control and local control of the removed metastases.
